# Clinical and molecular spectrum of P/Q type calcium channel Cav2.1 in epileptic patients

**DOI:** 10.1186/s13023-021-02101-y

**Published:** 2021-11-02

**Authors:** Elham Alehabib, Zahra Esmaeilizadeh, Sakineh Ranji-Burachaloo, Abbas Tafakhori, Hossein Darvish, Abolfazl Movafagh

**Affiliations:** 1 Student Research Committee, Department of Medical Genetics, School of Medicine, Shahid Beheshti University of Medical Sciences, Tehran, Iran; 2 Department of Medical Genetics, School of Medicine, Shahid Beheshti University of Medical Sciences, Tehran, Iran; 3 Iranian Center of Neurological Research, Neuroscience Institute, Tehran University of Medical Sciences, Tehran, Iran; 4 Neuroscience Research Center, Faculty of Medicine, Golestan University of Medical Sciences, Gorgan, Iran

**Keywords:** *CACNA1A*, Epilepsy, Calcium channels, Intellectual disability, Neurodevelopmental disorder, Brain imaging abnormalities

## Abstract

**Background:**

Epilepsy is a neurological disorder characterized by the potential to induce seizure and accompanied by cognitive, psychological, and social consequences. *CACNA1A* gene is a voltage-gated P/Q-type Cav2.1 channel that is broadly expressed in the central nervous system, and the pathogenic variants within this gene may be associated with the epileptic phenotype. In the present study, we collected clinical and molecular data related to epileptic patients with *CACNA1A* pathogenic variants and investigated possible meaningful relationship between age at onset, neurodevelopmental disorders, type of seizures, brain imaging abnormalities, genotype, and protein domains.

**Results:**

In our retrospective literature studies, from among 890 articles reviewed, a total of 90 individuals were related to epilepsy phenotype. Our findings showed that about 90 percent of patients have shown the first symptoms in childhood and teenage years and different types of neurodevelopmental disorders, such as intellectual disability, developmental arrest, and behavioral disorders, have been common findings for these patients. Further, a wide range of abnormalities have been observed in their brain imaging, and generalized seizures have been the most type of seizures in these patients. However, our data showed no specific genotype–phenotype correlation in epileptic patients with *CACNA1A* pathogenic alterations.

**Conclusions:**

Our study focused on epileptic phenotype in patients with *CACNA1A* pathogenic variants and showed a wide range of clinical and molecular heterogeneity with no specific genotype–phenotype correlation. It seems that incomplete penetrance, *de-novo* variants, and modifier genes are obstacles in predicting the clinical outcome.

**Supplementary Information:**

The online version contains supplementary material available at 10.1186/s13023-021-02101-y.

## Background

**Fig. 1:**
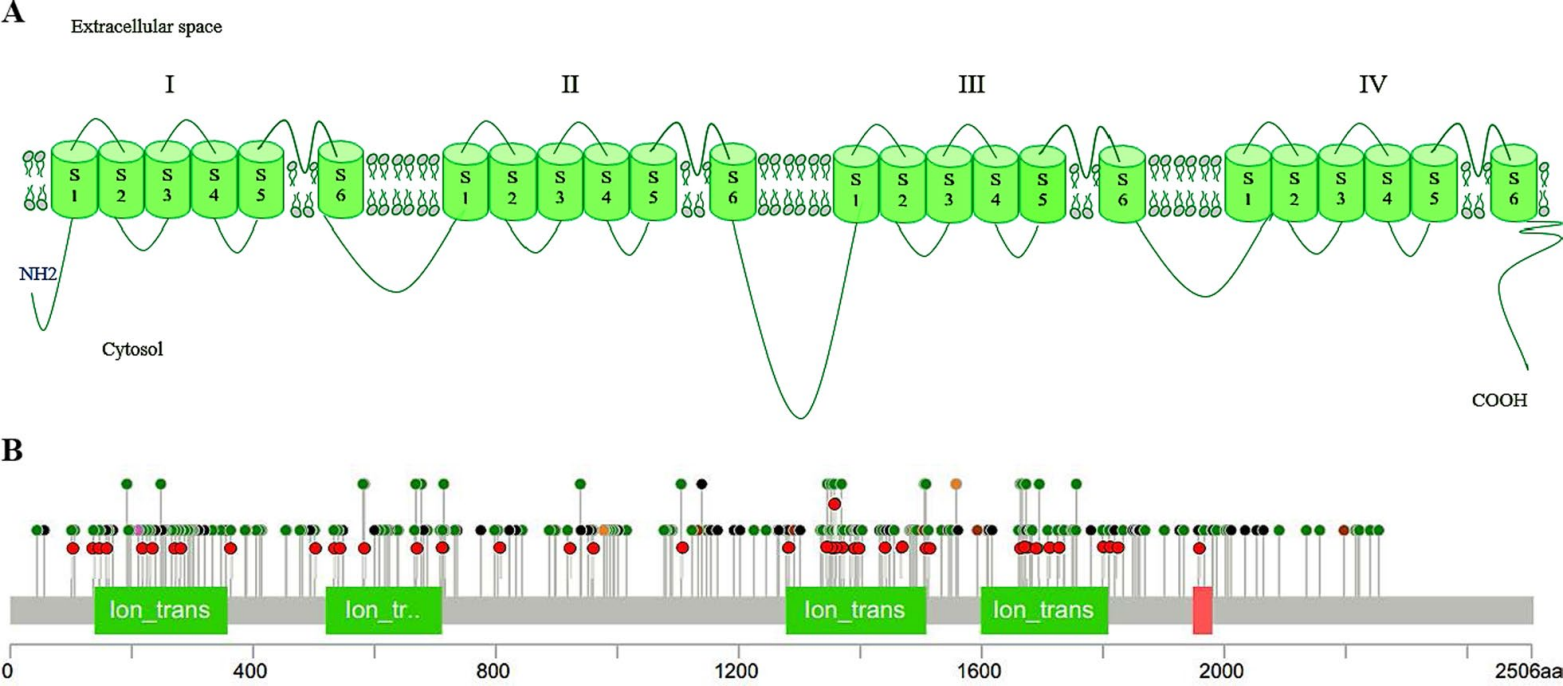
Schematic representation of CACNA1A protein (**A**) and distribution of pathogenic variants over the CACNA1A protein based on HGMD (red dots are related to epileptic patients) (**B**)

Voltage-Dependent Calcium Channels (VDCCs) are involved in the influx of calcium ions into excitable cells in response to membrane depolarization, and have different roles in calcium-dependent processes, including muscle contraction, neurotransmitter release, regulation of specific genes, and gene expression. Further, they are known to play a prominent role in the transmission of pain. VDCCs are composed of four subunits: alpha-1, beta, alpha2, and gamma [1, 2]. Alpha-1 has six isoforms and the *CACNA1A* gene, on the short arm of chromosome 19, encodes alpha-1A that is mainly expressed in neuronal tissue [3, 4]. Alpha1A subunit accounts for channel activity by pore-forming and consists of four repeated homologous domains (DI–DIV), each with six transmembrane regions (S1–S6) that make up two functional modules: a voltage sensor (S1–S4) and a Ca^2+^-selective pore (S5-P loop-S6) (Fig. 1A). The other subunits are considered as auxiliary and regulate this activity [1]. According to electrophysiological features, voltage-gated calcium channels are divided into two groups: high voltage-activated channels (L-, N-, P/Q-, and R- types) and low voltage-activated channels (T-type), and *CACNA1A* is in the P/Q type subgroup [5]. Voltage-gated P/Q-type Cav2.1 channels are broadly expressed in the central nervous system (CNS), and are especially expressed on cell bodies and dendrites of cerebellar purkinje cells and granule cells of the cerebellum. These channels are expressed on presynaptic terminals and mediate depolarization-induced calcium influx, which is tightly coupled with neurotransmitter release [6, 7]. Any pathogenic alterations in the *CACNA1A* gene, which lead to changed calcium influx, are associated with neurological disorders [4, 8–10]. For example, familiar hemiplegic migraine is related to increase in P/Q channel activity, while absence seizures and ataxia are related to decrease in P/Q channel activity. Besides, expansion of CAG repeats in the coding regions, which occurs in some transcript variants, can alter calcium influx and is associated with spinocerebellar ataxia 6 [4, 8–10]. Several case reports described epilepsy as a part of the clinical manifestation of disease in patients with *CACNA1A* mutations that can be the only symptom or can appear along with the other signs. Epilepsy is a neurological disorder characterized by its potential to cause seizures and its accompanying cognitive, psychological, and social consequences [11]. The estimated prevalence of epilepsy is about 6.4 in 1′000 people, and the standard mortality rate reported is around 2.3 and 2.6 for patients in developed and underdeveloped countries, respectively [12, 13]. About 20% to 30% of the epileptic cases happen as acquired factors, and genetic factors with 65% play a crucial role in causing this disease [14]. More than 100 genes are involved in developing epilepsy phenotype, and there have been several reports of the *CACNA1A* gene. In the present study, we made an attempt to collect clinical and genetic data related to epileptic patients with *CACNA1A* pathogenic variants and took a closer look to determine the possible relationship between age at onset, phenotype, type of seizure, neuroimaging finding, and protein domain.

## Results

In our retrospective literature study, we reviewed more than 890 articles among which 90 individuals were related to epilepsy or recurrent seizures and had confirmed pathogenic variants. Below we take a closer look at the data that led to our findings.

### Age at onset

**Fig. 2:**
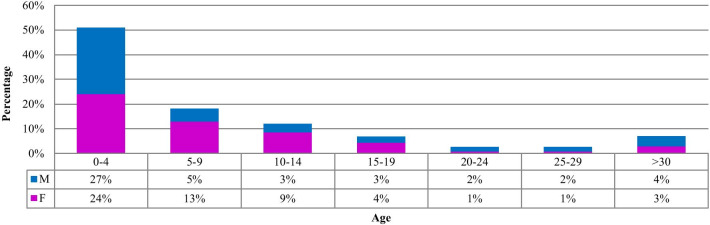
Age at onset distribution

Age at onset distribution (Fig. 2) showed that about 50 percent of cases were classified under four years of age, and about 90 percent of all cases had manifested their first symptoms in childhood or teenage years (before adulthood). In other words, this gene can affect developmental processes. Patients with late-onset have a mild phenotype, which does not usually have a remarkable effect on their life. Age at onset is not shown to have any meaningful connection with involved domains in the CACNA1A protein domain. Also, each pathogenic alteration has shown different ages at onset, even in members of a single family.

### Neurodevelopmental disorders

Based on our investigations, in patients with *CACNA1A* pathogenic variants and epileptic phenotype, neurodevelopmental disorders are the most common findings which are divided into three categories, namely intellectual disability, developmental arrest, and behavioral disorders, although in some reports epilepsy or seizures were the only remarkable findings. Also, intelligence quotient in patients showed a wide range, i.e. borderline (learning disabilities), mild, moderate, severe, and profound. Furthermore, in these patients, psychiatric comorbidities, as behavioral disorders, were diagnosed, including autism, atypical social interactions, and Attention Deficit Hyperactivity Disorder (ADHD).

### Type of seizure

**Fig. 3:**
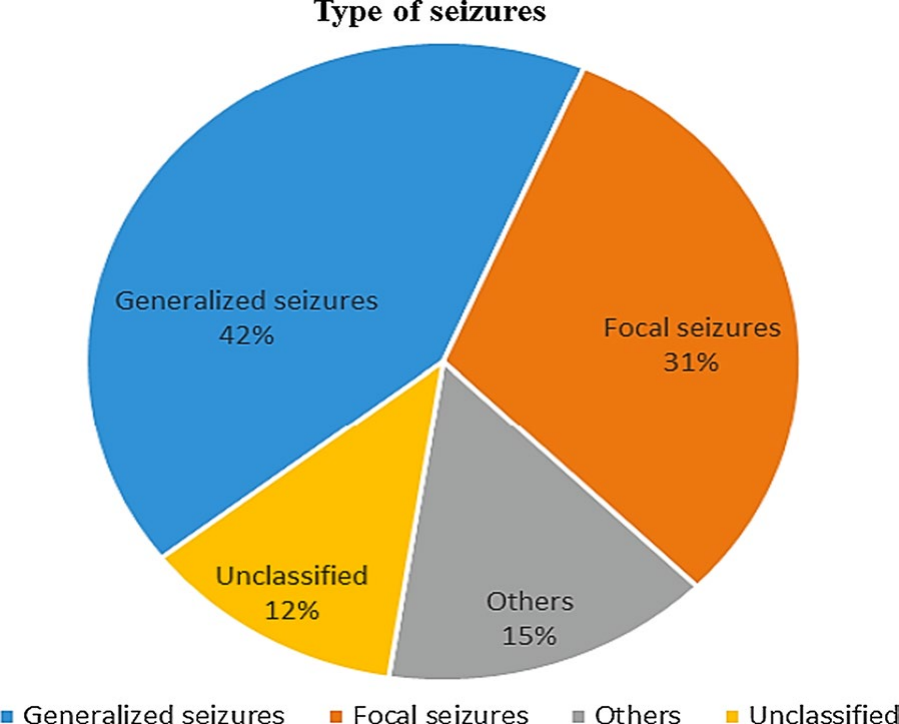
Type of seizures

Investigation in clinical data in patients with *CACNA1A* pathogenic variants and epilepsy as an only or one of the symptoms have shown that different types of seizures may occur in these patients. Affected family members may not develop the same seizure, or seizure may not be a common symptom among them, meaning that affected family members with the same pathogenic variant may or may not have seizures at all. Figure 3 shows the types and the percent of seizures in patients with *CACNA1A* pathogenic alterations (all affected members of each family included). Based on this pie chart, generalized seizures are the most frequent type of seizures in these patients and include generalized tonic–clonic seizures, absence seizures, and myoclonic astatic seizures. The type of seizures in patients is not shown to have any meaningful connection with pathogenic alterations in each part of the CACNA1A protein domain. Also, each pathogenic variant can present different types of seizures, even in members of a single family.

### Brain imaging abnormalities

**Fig. 4:**
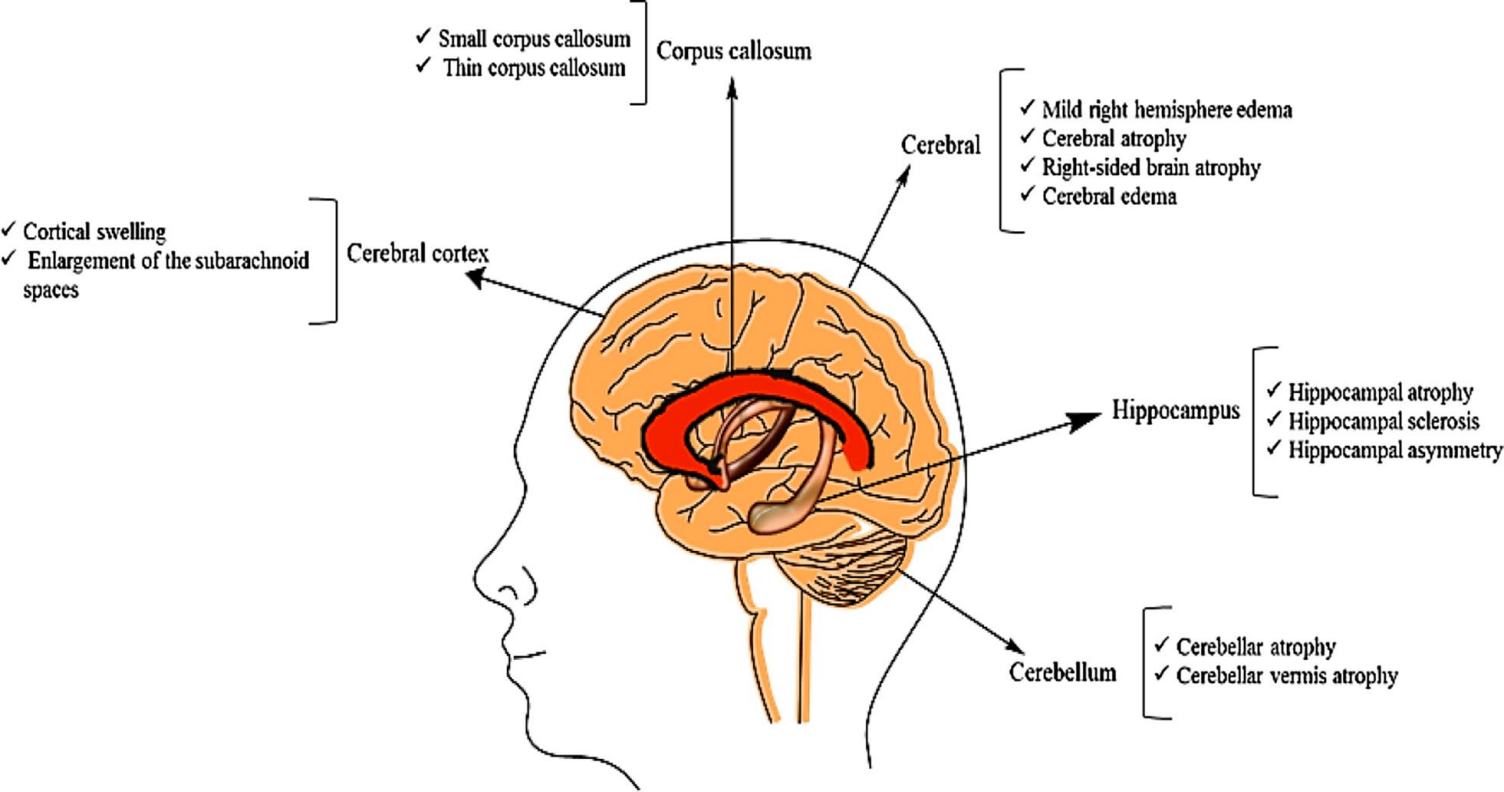
The spectrum of imaging findings in epileptic patients with *CACNA1A* pathogenic variants

Generally, brain imaging findings have shown a wide range of abnormalities in the cerebral cortex, cerebral, cerebellar, hippocampus, and corpus callosum (Fig. 4), although in some reports brains showed no abnormality, at least at the time of publishing the articles. Brain imaging findings and pathogenic variant positions were not observed to have any significant relationship in the protein domains.

### Bioinformatics analysis

**Fig. 5:**
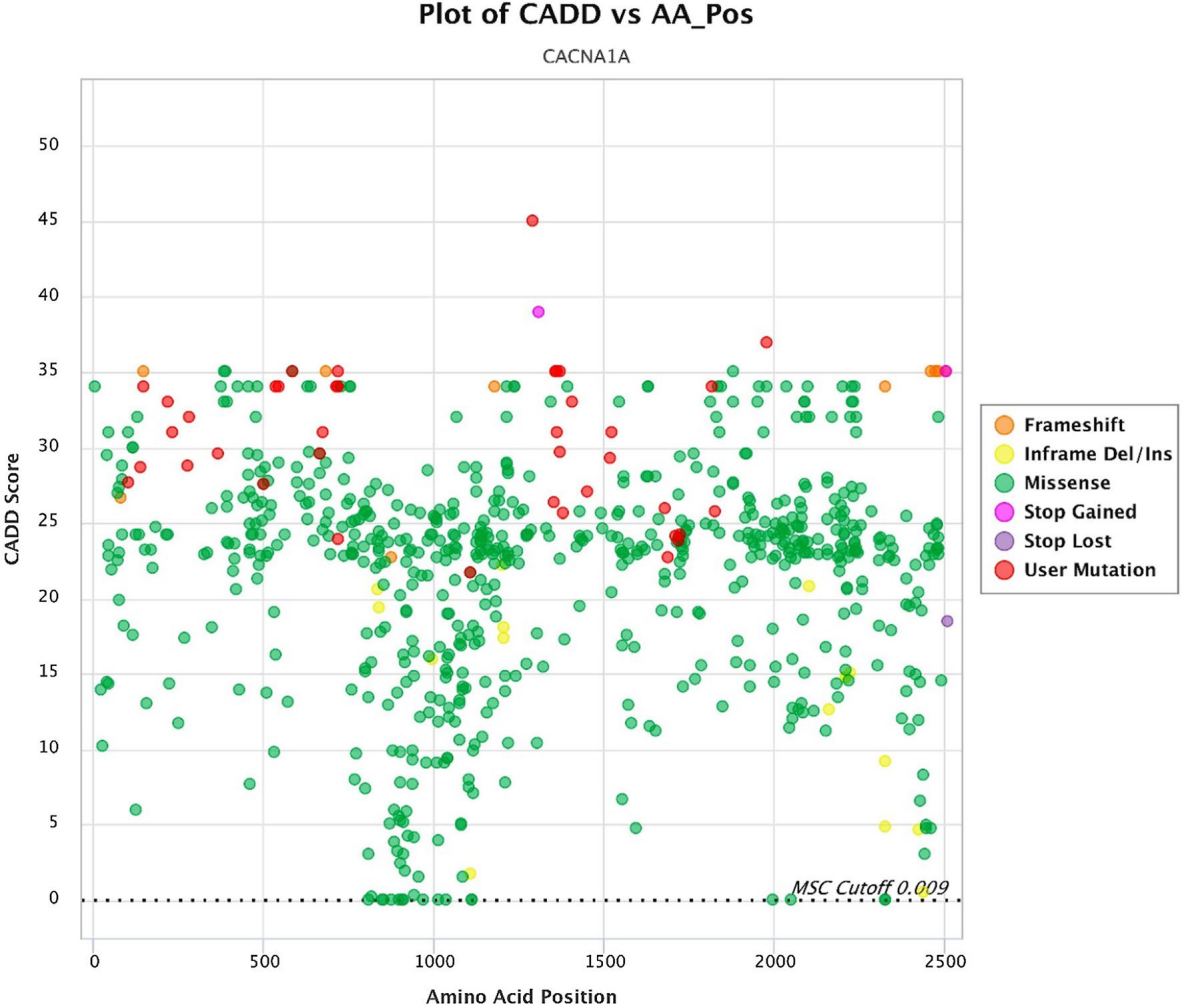
Plot of CADD versus AA_Pos (red dots: pathogenic variants in epileptic patients)

**Fig. 6:**
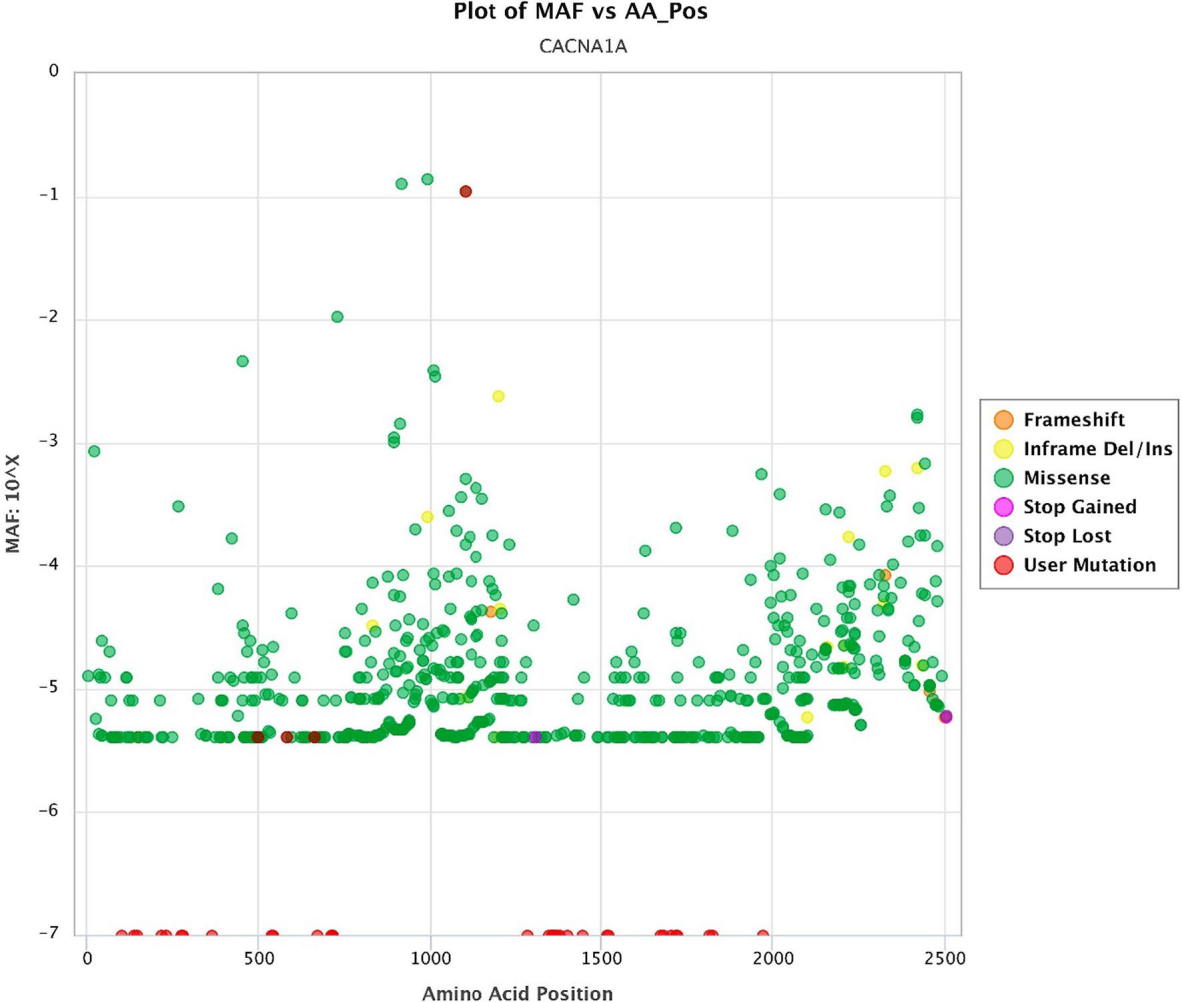
Plot of MAF versus AA_Pos (red dots: pathogenic variants in epileptic patients)

**Fig. 7:**
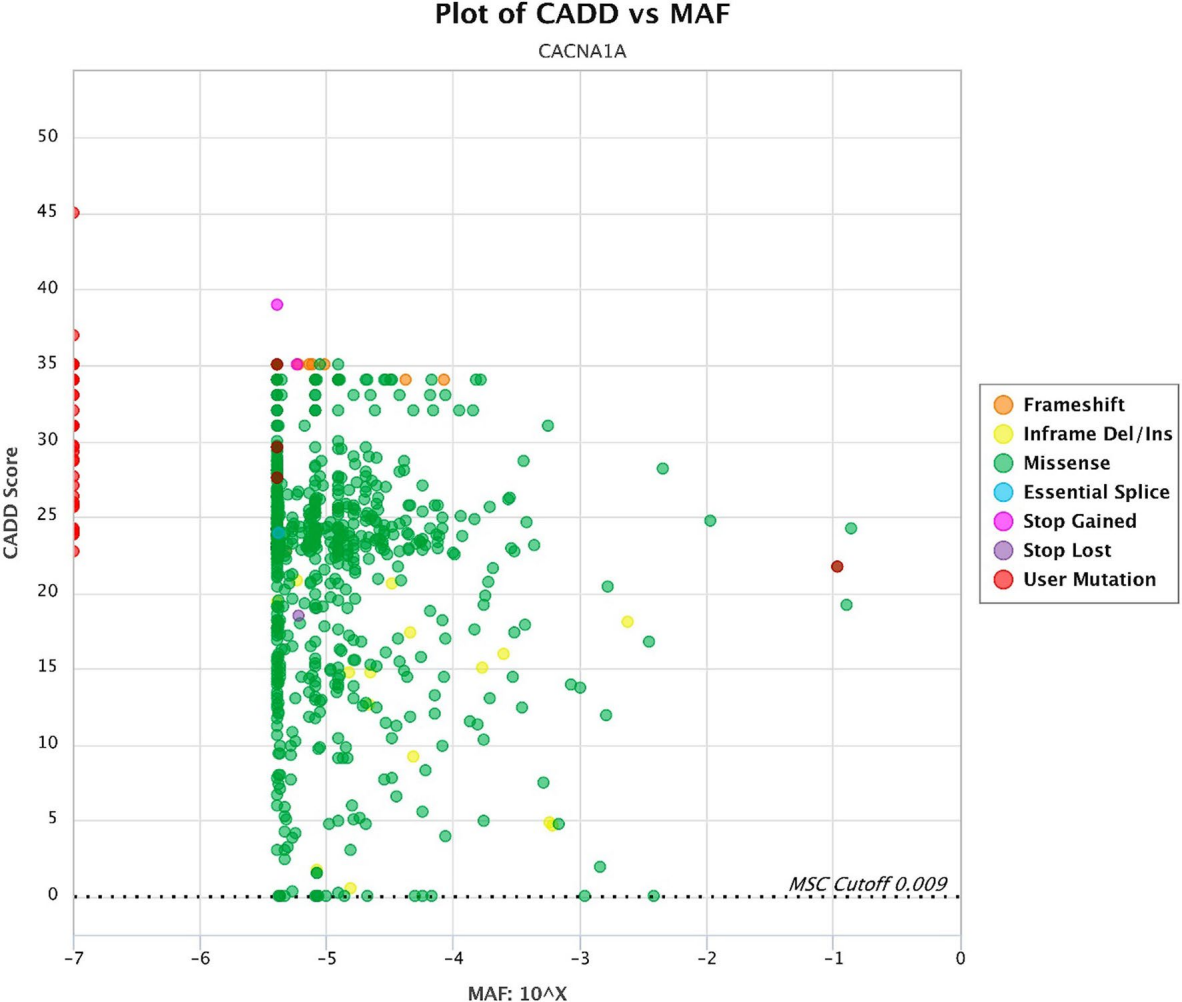
Plot of CADD versus MAF (red dots: pathogenic variants in epileptic patients)

To visualize minor allele frequencies and damage prediction scores of human genetic variation, we used Popviz, an integrative and interactive webserver [15]. Although there are several mutation damage prediction tools available, PopViz is particularly useful in the investigations of sequencing data in a gene and variants by supporting or refuting the reasonability of the candidate gene and by selecting and prioritizing the candidate variants for experimental testing. This webserver provides visualization of the variants based on minor allele frequencies from gnomAD database, damage prediction scores from multi approach (CADD, EIGEN or LINSIGHT), and amino-acid positions and protein domain. Accordingly, the *CACNA1A* graphs illustrate the common type of genetic change in *CACNA1A* gene (frameshift, inframe Del/Ins, missense, stop gained, stop lost), frequency of variants (both pathogenic and non-pathogenic), and distribution of the variants in different regions of the protein (Figs. 5, 6, 7). Altogether, these data can be used in the interpretation of the variants and to further understand how they are related to the disease.

Figures 1B and 5 illustrate *CACNA1A* variants distribution throughout CACNA1A protein and their CADD scores based on HGMD and gnomAD. Individuals in gnomAD are (supposedly) healthy, and the presence of several common variants (MAF > 10^−3^) with a CADD score of > 20 in the gnomAD data highlights the difficulties when interpreting *CACNA1A* variation in patients since these variants are not likely to cause disease. Also, the scattering distribution of variants could be used to identify mutational "hot spots" and locations within the gene that are rather prone to benign variations. As Figs. 1B and 5 illustrate, the most part of the gene is prone to nucleotides alteration and effect.

MAF versus amino acid position’s plot illustrates a large part of variants that cause changes almost throughout the gene have low frequency (less than 10^–4^) (Fig. 6) which, along with de novo variants, incomplete penetration, and development of mild diseases have brought about challenges in the interpretation of this variant. Consequently, CADD versus MAF plot (Fig. 7) illustrates combination of the two plots above without considering amino acid position, which can help our overall insight for variant analysis in the gene. In genes that do not cause fatal, severe, and congenital diseases and have incomplete penetration and different onset ages, or show a spectrum of clinical signs, pathogenic variants may be more frequent. Therefore, in the face of such genes, variants must be examined more carefully.

## Discussion

In the current study, we investigated reported families with epilepsy or recurrent seizures and *CACNA1A* pathogenic variants and then, looked for any relationship between age at onset, neurodevelopmental disorders, type of seizures, brain imaging abnormalities, genotype, and protein domains. Our findings (Additional file 1) revealed that (1) about 90 percent of patients have shown the first symptoms in childhood and teenage years, (2) neurodevelopmental disorders have been common findings in these patients, including intellectual disability, developmental arrest, and behavioral disorders, (3) generalized seizures have been the most frequent type of seizures in patients, (4) a wide range of abnormalities have been observed in brain imaging, which involved cerebral cortex, cerebral, cerebellar, hippocampus, and corpus callosum, and (5) there is no specific genotype–phenotype correlation in epileptic patients with *CACNA1A* pathogenic alterations.

*CACNA1A* gene has 47 exons that code for a protein with 2506 amino acids, and its protein consists of four (I–IV) repeated domains. Each domain contains six (S1–S6) transmembrane regions with a voltage sensor (S1–S4) and a pore region (Fig. 1A). Intracellular loop between domains II and III have interactions with receptors of G-protein-coupled. Also, there are important motifs that interact with the soluble N-ethylmaleimide-sensitive factor attachment protein receptor (SNARE). These interactions have an essential role in vesicular docking that occur before exocytosis [16]. As shown in the previous studies, pathogenic variants in *CACNA1A* gene sequences, especially in this region, could alter the conformation of the CACNA1A protein [17]. In epileptic patients with *CACNA1A* mutations, all domains have shown pathogenic variants, but it seems that the intracellular loop between domains II and III and domain III is more than those of others. Also, C-terminal and N-terminal have essential roles in channel gating regulating processes. Presently, more than 280 disease-causing variants in *CACNA1A* have been reported in the Human Gene Mutation Database (HGMD). More than half (68.05%) of the variants are missense or nonsense mutations, 20.1% are gross and small deletions, 5.20% are small insertions, and the remaining are splicing substitution (4.51%), expansion repeat (1.73%), and complex rearrangement (0.34%) mutations. Most of the variants in the *CACNA1A* gene are point mutations, specifically missense mutations, and distributed throughout the gene. Also, most of these variants are rare (frequency: less than < 10^–4^) and have a high CADD score (> 20). Due to the type of mutations and their function (gain or loss), *CACNA1A* pathogenic variants have been associated with numerous neurological disorders, including Developmental and Epileptic Encephalopathy (DEE), episodic ataxia, type2 (EA2), familial hemiplegic migraine (FHM), familial hemiplegic migraine with progressive cerebellar, and spinocerebellar ataxia6 (SCA6). FHM is independently associated with the risk of stroke and epilepsy, and probably because of cortical spreading depression that occurs in FHM, migraine with aura has a double risk. Furthermore, when FHM is due to a CACNA1A protein defect, it will be more significant as it can, by itself, lead to ion homeostasis, increase in neuronal excitability, and decrease in threshold for CSD. Up to now, some pathogenic variants in *CACNA1A* have been reported, which are in association with stroke and epilepsy. These variants showed gain of function, and in some cases, the same mutation was reported along with FHM [18]. Hence in the molecular examination for early age at onset of stroke (childhood stroke), *CACNA1A* should be considered [19, 20]. Although there are some reports about the other related phenotypes with low counts, including benign paroxysmal torticollis (BPT), tremor, which expand the spectrum of clinical phenotypes in this gene, the main reasons for this spectrum of the disorder have not been understood yet. Previous studies [20–22] have shown that EA2 usually happens as a result of non-sense mutations or deletions and loss of function, FHM and infantile epilepsy with myoclonus happen probably as a result of missense mutations and loss of function in P/Q type calcium channel activity, and DEE happens as a result of both gain of function and loss of function. In truncating protein, the probable explanation of its mechanisms is truncated fragment binding to regulatory elements and polyQ structure [21–23]. A previous study showed that knocking out GABRG2 (Gamma-Aminobutyric Acid Type A Receptor Subunit Gamma2) leads to a decrease in the expression of *CACNA1A*, which promotes Generalized epilepsy with febrile seizures plus (GEFS+) disease and increases temperature leading to gradual decrease in *CACNA1A* expression and contributes to epileptogenesis [24, 25]. Ankyrin B (AnkB) is a scaffold for various ion channels (including *CACNA1A*) and expresses anywhere, including the brain. *CACNA1A* is an interactor for AnkB and has a crucial role in neuronal function. Intracellular and surface levels of Cav2.1 (*CACNA1A*) are regulated by AnkB and change depending on the type of nucleotide acid alteration in AnkB. Therefore, *CACNA1A* and AnkB have a significant role in health or disease and neuronal function [26]. Patients with BPT of infancy have shown positive family history with various clinical presentations, such as FHM without the symptom of BPT [9, 27–29]. Besides, patients with intention tremor or head tremor, as a sign of cerebellar dysfunction, have shown positive family history in other neurological problems, such as ataxia or epileptic seizure and migraine in their family [30, 31], meaning that these rare clinical features are not separated. Previous studies have also shown the role of the *CACNA1A* gene in causing epilepsy. Rossignol and his colleagues demonstrated a relationship between different types of seizures and several epileptic disorders. They showed that *CACNA1A* loss of function reduces synaptic efficiency from cortical pyramidal cells and can prevent motor seizure. They concluded that pathogenic variants that impair cortical interneuron function can lead to severe epileptic syndrome, and pathogenic variants that affect synaptic transmission lead to a mild phenotype [32]. Different types of seizure were reported related to *CACNA1A*, which include generalized seizures (absence seizures, myoclonic-astatic, generalized tonic–clonic), focal seizures (complex partial seizure, atypical focal seizures, focal myoclonic, focal (tonic or clonic) seizures), and, with lower frequency, the other types of seizure, including atypical febrile seizure, status epilepticus, and febrile seizure [33–36]. Our findings showed that the common types of seizures in epileptic patients with *CACNA1A* mutations are generalized seizures (absence seizures, myoclonic-astatic, and generalized tonic–clonic) and focal seizures (complex partial seizure, atypical focal seizures, focal myoclonic seizures, and focal (tonic or clonic) seizures), respectively (Fig. 3). Several studies confirmed that CAG repeat in coding or non-coding regions of some genes sequences may cause a group of neurodegenerative disorders with autosomal dominant inheritance, including huntington's disease (HD), some spinocerebellar ataxia [1–3, 6, 7], machado-joseph disease (MJD), dentatorubropallidoluysian atrophy (DRPLA), and spinobulbar muscular atrophy [37, 38]. The C-terminus of *CACNA1A* has a significant role in the correct function, folding, and processing. Pathogenic CAG repeats in this region can lead to a dominant-negative mechanism between *CACNA1A* wild type and mutant alleles and cause disturbance of surface expression of wild type CACNA1A protein [39]. Functional analysis in the cell line model (SH-SY5Y neuroblastoma cell line) revealed that CAG repeats of pathogenic expansion in the C-terminus of the *CACNA1A* gene probably lead to activation of apoptosis factors (Bcl-2/Bax, caspase, and PARP) and induce cell apoptosis in nerve cells. This finding can provide new insights for the treatment of Progressive Myoclonic Epilepsy (PME) [21]. Ataxia, myoclonus seizures, and progressive cognitive impairment are some features that are usually present in PME patients [40]. Previous studies have confirmed that expansion of CAG repeat in the *CACNA1A* gene is pure, and repeat interruption does not influence age at onset or disease progression [41]. Most of the mutations in *CACNA1A* are de novo and a previous study showed a specific gene set that is regulated by FMRP wherein de novo mutations were normally observed (*P* < 10^–8^) [42]. Furthermore, FMRP has direct interaction with *CACNA1A*, and so it can probably explain the cause of psychiatric disorder in the clinical spectrum related to this gene [42, 43]. Detailed examination of epileptic patients with *CACNA1A* mutations has shown that a handful of them have been showing psychiatric problems including atypical social interactions, ADHD, and autism. The functional investigation on the effect of *CACNA1A* loss of function has shown that postnatal dysfunction is related to the clinical findings. Brain atrophy, cognitive disorders, and psychiatric disorders are some side effects that are usually observed in these patients [44]. With respect to several types of alterations in the *CACNA1A* gene, which lead to different diseases, and a diverse range of symptoms that can have an overlap, both repeat expansions and nucleotide alteration should be evaluated [45]. Besides, functional analysis is required to assign pathogenicity and molecular mechanism by which each variant affects the *CACNA1A* channel [46–49]. Previous studies have also shown broad clinical heterogeneity and incomplete penetrance, even in intra-family cases. These challenges lead to difficulties in accurate genetic counseling and determine the pathogenicity of variants related to *CACNA1A* [50]. *CACNA1A* variants and absence seizures are related to each other, and this issue has been shown in rodents and humans. In addition, the role of *CACNA1A* variants in developing absence seizures in dravet syndrome has been reported [51]. Consequently, the *CACNA1A* gene can be proposed as a modifier gene [51, 52]. Moreover, functional studies related to *CACNA1A* variants were carried out in the mouse model to find the effect of the deleterious variant on the *CACNA1A* gene [51]. Given that Calcium channels express in several organs, including the cerebellum, thalamus, cortex, and hippocampus, a possible explanation for clinical heterogeneity and different presentation of this disease is different localization of these channels [50, 53]. The probable explanation for incomplete penetrance or reduced penetrance in patients with pathogenic variants in the gene is that pathogenic alleles in ion channels protein can lead to therapeutic combination and decrease or mask clinical presentation expressivity. This can also be a probable explanation about sporadic cases, confusing genotype–phenotype relationship, or even some complexities observed in Mendelian pedigree [54]. While *CACNA1A* usually considers a young-onset disease, some reports have shown that pathogenic variants in this gene lead to late-onset diseases, such as episodic ataxia 2 (EA2) and progressive cerebellar ataxia (PCA) [55].

## Conclusion

In the present study, we collected clinical and genetic data related to epileptic patients with *CACNA1A* pathogenic variants and investigated any meaningful relationship between age at onset, neurodevelopmental disorders, type of seizures, brain imaging abnormalities, genotype, and protein domains. Our findings showed that about 90 percent of patients have shown the first symptoms in childhood and teenage years and different types of neurodevelopmental disorders, such as intellectual disability, developmental arrest, and behavioral disorders, have been common in these patients. Further, a wide range of abnormalities have been observed in their brain imaging, and generalized seizures have been the most type of seizures in these patients. However, our data showed no specific genotype–phenotype correlation in epileptic patients with *CACNA1A* pathogenic alterations.

## Methods

### Study selection

Included cases were retrieved from the available databases using the following keyword: *CACNA1A*. In the next step, papers with data on genetics and epileptic phenotype were selected and carefully examined for collecting the information on sex, age at onset, neurological phenotype and cognitive status, type of seizure, neuroimaging finding, and genotype. Subsequently, we looked more closely to find any possible connection between the extracted information and the protein domains.

### Bioinformatics analysis

We used PopViz Integrative and interactive webserver to visualize population genetics (based on gnomAD) and mutation damage prediction scores of human gene variants to illustrate *CACNA1A* variants based on amino acid position, minor allele frequency (MAF), and Combined Annotation Dependent Depletion (CADD) score [15]. The study was approved by Shahid Beheshti University of Medical Sciences, Tehran.

## Supplementary Information

**Additional file 1.** Clinical and molecular data related to epileptic patients with *CACNA1A* pathogenic variants.

## Data Availability

Not applicable.
